# Neddylation is essential for malaria transmission in *Plasmodium berghei*

**DOI:** 10.1128/mbio.00232-24

**Published:** 2024-02-27

**Authors:** Bandita Nayak, Plabita Paul, Satish Mishra

**Affiliations:** 1 Division of Molecular Microbiology and Immunology, CSIR-Central Drug Research Institute, Lucknow, Uttar Pradesh, India; 2 Academy of Scientific and Innovative Research (AcSIR), Ghaziabad, Uttar Pradesh, India; The George Washington University Milken Institute of Public Health, Washington, USA

**Keywords:** *Plasmodium*, posttranslational modification, neddylation, Nedd8, malaria transmission

## Abstract

Neddylation is a type of posttranslational modification known to regulate a wide range of cellular processes by covalently conjugating the ubiquitin-like protein Nedd8 to target proteins at lysine residues. However, the role of neddylation in malaria parasites has not been determined. Here, for the first time, we showed that neddylation plays an essential role in malaria transmission in *Plasmodium berghei*. We found that disruption of Nedd8 did not affect blood-stage propagation, gametocyte development, gamete formation, or zygote formation while abolishing the formation of ookinetes and further transmission of the parasites in mosquitoes. These phenotypic defects in Nedd8 knockout parasites were complemented by reintroducing the gene that restored mosquito transmission to wild-type levels. Our data establish the role of *P. berghei* Nedd8 in malaria parasite transmission.

**IMPORTANCE:**

Neddylation is a process by which Nedd8 is covalently attached to target proteins through three-step enzymatic cascades. The attachment of Nedd8 residues results in a range of diverse functions, such as cell cycle regulation, metabolism, immunity, and tumorigenesis. The potential neddylation substrates are cullin (CUL) family members, which are implicated in controlling the cell cycle. Cullin neddylation leads to the activation of cullin-RING ubiquitin ligases, which regulate a myriad of biological processes through target-specific ubiquitylation. Neddylation possibly regulates meiosis in zygotes, which subsequently develop into ookinetes. Our findings point to an essential function of this neddylation pathway and highlight its possible importance in designing novel intervention strategies.

## OBSERVATION

Protein neddylation is a biochemical process composed of the enzymes E1 [Nedd8 activating enzyme (NAE)], E2 (Nedd8 conjugating enzyme), and E3 (ligase), and the deconjugating enzymes required for the deconjugation of neural precursor cell expressed developmentally downregulated 8 (Nedd8) to a protein (1). Nedd8 activates the largest ubiquitin E3 ligase family, cullin-RING ubiquitin ligases (CRLs) (2). The most well-studied substrate of Nedd8 is cullin, and the most commonly reported Nedd8-associated processes involve the neddylation of cullin-RING E3 ubiquitin ligases (2), Skp1-Cullin-F-box protein (SCF) E3 ubiquitin ligase complex, and APC/C (anaphase-promoting complex/cyclosome). CRLs, SCFs and APCs are key components in controlling the cell cycle (3, 4) and DNA damage repair (5). Deneddylation of the noncullin substrate is carried out by NedP1/Den1/SENP8 (6–8). The apicomplexan UCHL3 and *Plasmodium falciparum* UCH54 retain dual specificity for deubiquitinating/deNeddylating activity (9–11).

There are two unique processes of mitosis and one period of meiosis during the *Plasmodium* life cycle (12). Mitotic division occurs during male gametogenesis, during which the octoploid stage occurs, followed by chromosome condensation and nuclear budding into male gametes (13). Meiosis occurs in diploid cells, and the zygote is the only diploid stage in the parasite (14). During meiosis, DNA is duplicated to produce a tetraploid cell through six morphological stages (I–VI) over a period of 24 h. The master regulators of these stages are not fully understood; however, the roles of several proteins, such as centrins (15), cell-division cycle protein 20, CDC20 homolog 1 (16), the anaphase-promoting complex (APC) (17), and condensin core subunits (SMC2/SMC4) (18), have been implicated. During male gamete development, the haploid nucleus, associated microtubule-organizing center, and flagellum form eight motile microgametes through exflagellation (19).

Bioinformatics studies have identified Nedd8 and several pathway components in protozoan parasites. *P. falciparum* Nedd8 complements the *Saccharomyces cerevisiae* Nedd8 homolog Rub1 and interacts with cullins (20). Recently, Karpiyevich et al. demonstrated that the deubiquitinating enzyme PfUCH37-mediated activity of Nedd8 was dispensable for parasite viability. However, its deubiquitinating activity is essential for the viability of *P. falciparum* blood stages (21). The study of neddylation in the protozoan parasite *Trypanosoma brucei* revealed atypical features of the parasite Nedd8 compared to those of higher eukaryotes (22). In this study, we showed that Nedd8 is dispensable in blood stages. We found that male and female gametes of the Nedd8 knockout (KO) parasite fused to form zygotes; however, the zygotes failed to differentiate into ookinetes.

**Fig 1:**
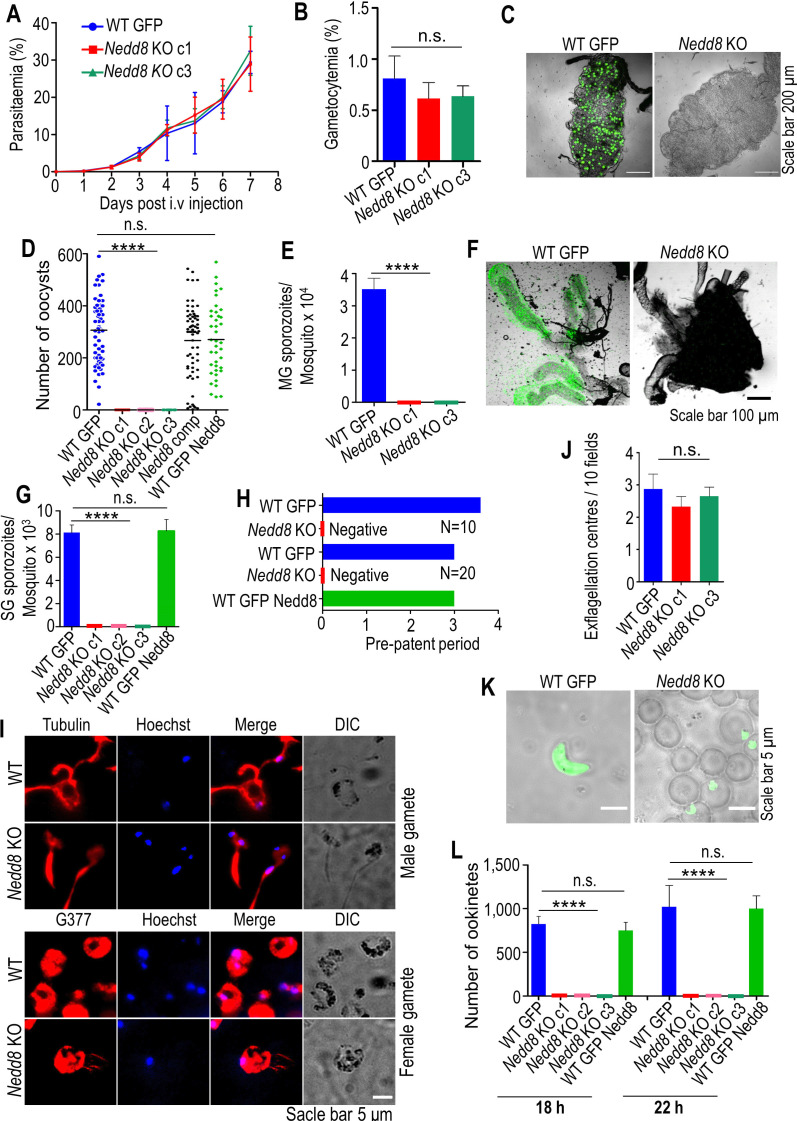
Neddylation is essential for malaria transmission. (**A**) *Nedd8* KO parasites grow normally during asexual blood-stage propagation. The data are presented as the mean ± SEM from three independent experiments; no significant difference was detected (*P* = 0.96) by one-way ANOVA. (**B**) Gametocytemia in *Nedd8* KO parasites was similar to that in WT GFP-expressing parasites. The error bars show the means ± SEMs of three independent experiments; there were no significant differences in the c1 (*P* = 0.3179) or c3 (*P* = 0.3802) levels according to one-way ANOVA. (**C**) The WT parasites produced GFP-expressing oocysts, whereas no GFP signals were observed in the *Nedd8* KO parasites. (**D**) The midgut was dissected, and the oocyst number per mosquito was counted. No oocyst development was observed in *Nedd8* KO parasites. The data are presented as the mean ± SEM from three independent experiments. *P* < 0.0001 was considered to indicate a significant difference (one-way ANOVA). WT GFP, Nedd8 comp, and WT GFP Nedd8 did not significantly differ (*P* = 0.1820). (**E**) Midgut sporozoite count; no sporozoites were observed in KO parasites. (**F**) Salivary glands showing GFP expression; no GFP expression was observed in KO parasites. (**G**) Salivary gland sporozoite count; no sporozoites were observed in the KO parasites. (**H**) Infection in C57BL/6 mice inoculated with *Nedd8* KO parasites (five mice per group). Mosquitoes (*n* = 10 or 20) were allowed to probe for blood meal, and the appearance of parasites in the blood was observed by making a Giemsa-stained blood smear. (**I**) Normal formation of gametes in *Nedd8* KO parasites. (**J**) The exflagellation centers in *Nedd8* KO parasites were similar to those in WT GFP parasites. Error bars show the mean ± SEM from three independent experiments; no significant difference was detected (*P* = 0.2514) by one-way ANOVA. (**K**) Mosquitoes were dissected 18 and 22 h postfeeding to observe ookinetic development under a fluorescence microscope. No ookinetes were observed in *Nedd8* KO parasites, whereas WT GFP parasites exhibited normal ookinete development. (**L**) Ookinete number; no ookinetes were observed in KO parasites. The data from 50 mosquitoes per group are presented as the mean ± SEM of three independent experiments; significant differences (****P < 0.0001); one-way ANOVA. There was no difference between the WT GFP and the WT GFP Nedd8 parasites (*P* = 0.3847, 18 h and *P* = 0.9046, 22 h). ANOVA, analysis of variance; n.s., not significant; SEM, standard error of the mean.

To determine the function of Nedd8 in the *P. berghei* life cycle*,* the gene was disrupted using double crossover homologous recombination (see Fig. S1A at https://doi.org/10.6084/m9.figshare.25224743.v1). For this purpose, two homologous fragments, F1 and F2, were amplified and cloned into the pBC-GFP-hDHFR:yFCU vector. The vector was linearized and transfected into *P. berghei* ANKA schizonts, and successful transfection was confirmed by observing drug-resistant green fluorescent protein (GFP)-expressing parasites (see Fig. S1B at https://doi.org/10.6084/m9.figshare.25224743.v1). Genomic DNA was isolated from the drug-resistant GFP-expressing parasites, and diagnostic PCR was performed, which yielded bands of 1.2 and 0.85 kb, confirming the correct 5′ and 3′ site-specific integrations, respectively (see Fig. S1C at https://doi.org/10.6084/m9.figshare.25224743.v1). A Southern blot was carried out to further confirm the genomic DNA manipulation. The DNA was digested with *EcoR*I and hybridized with a 5′ probe, which yielded bands of 8.6 and 3.6 kb in the wild type (WT) and *Nedd8* KO strains, respectively, confirming the *Nedd8* locus modification (see Fig. S1D at https://doi.org/10.6084/m9.figshare.25224743.v1). Finally, the absence of the *Nedd8* ORF in *Nedd8* KO parasites was confirmed by PCR (see Fig. S1E at https://doi.org/10.6084/m9.figshare.25224743.v1). The knockout parasites were also complemented for functional restoration (Fig. S1F and G at https://doi.org/10.6084/m9.figshare.25224743.v1). Another control parasite line was generated by knocking in a Nedd8 expression cassette into WT GFP parasites (see Fig. S2A at https://doi.org/10.6084/m9.figshare.25224743.v1). The correct site-specific integration was confirmed by diagnostic PCR (see Fig. S2B at https://doi.org/10.6084/m9.figshare.25224743.v1). To determine the effect of gene deletion on blood-stage development, equal numbers of WT GFP and KO iRBCs were injected into Swiss mice (five mice per group). Parasite growth in both groups was monitored daily by observing Giemsa-stained blood smears. We found no difference in the growth of *Nedd8* KO parasites compared to that of WT GFP parasites (Fig. 1A). Next, we monitored gametocytemia on day 3 postinjection and found that it was normal in both groups (Fig. 1B).

Next, to analyze the *Nedd8* KO phenotype in mosquito and mammalian hosts, female *A. stephensi* mosquitoes were allowed to feed on mice infected with either WT GFP or KO parasites. On day 14 postfeeding, the mosquitoes were dissected, and oocyst development in the midgut was observed. We found that the oocyst pattern, number, and sporulation status were normal in the WT GFP parasites, whereas no oocysts or oocyst-associated sporozoites were observed in the *Nedd8* KO parasites (Fig. 1C, D and E). Despite the lack of oocysts in *Nedd8* KO parasites, we checked the salivary glands for the presence of any sporozoites. Furthermore, we allowed Nedd8 KO-infected mosquitoes to feed on mice to evaluate their transmission ability. We did not observe GFP fluorescence or associated sporozoites in the salivary glands of the KO-infected mosquitoes (Fig. 1F and G). Blood-stage parasites were not detected in mice bitten with KO-infected mosquitoes (Fig. 1H; see Table S1 at https://doi.org/10.6084/m9.figshare.25224743.v1). Stages preceding oocyst formation were analyzed to determine the stage-specific defects in *Nedd8* KO parasites. The egress of gametocytes from host Red Blood Cells (RBCs) upon activation is a very dynamic process, and we found normal activation of female and male gametes (Fig. 1I). Male gametes were counted by observing the formation of exflagellation centers, which was comparable to that of WT GFP (Fig. 1J). To analyze the role of *Nedd8* beyond gametes, ookinete formation in *Nedd8* KO parasite-infected mosquitos was observed at 18 and 22 h after feeding, but ookinetes were not observed in *Nedd8* KO parasites (Fig. 1K and L). These results suggest that neddylation is required for ookinete formation and further malaria transmission.

**Fig 2:**
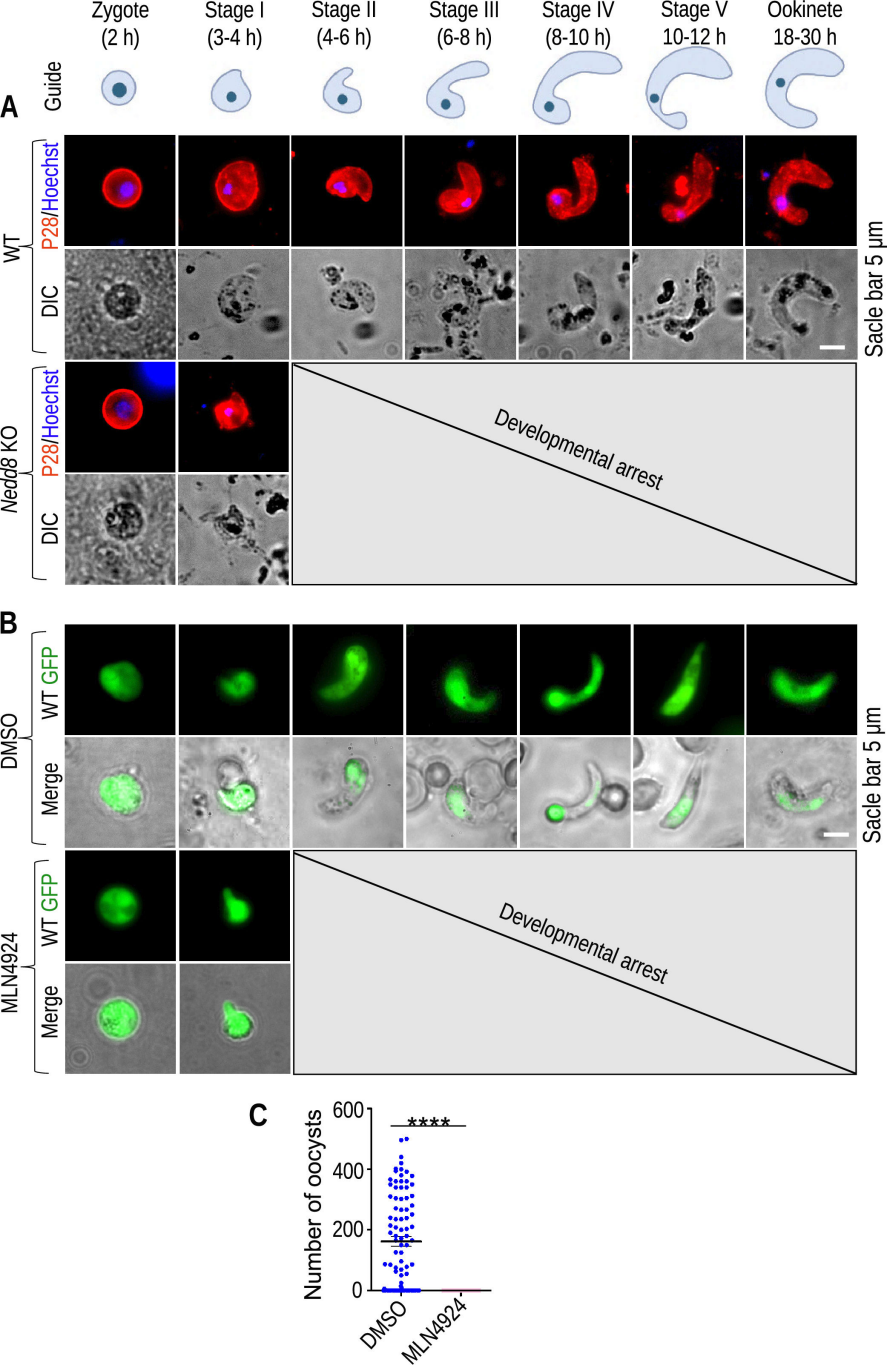
Nedd8 is essential for zygote-to-ookinete differentiation (**A**) Immunofluorescence assay (IFA) showing zygote-to-ookinete development *in vitro*. Schematic showing the morphological changes that occur during zygote-to-ookinete differentiation. A lack of *Nedd8* arrested parasite development during the stage I–II transition. Different morphological shapes were recorded in three independent experiments. (**B**) Morphologies of untreated and treated WT GFP parasites. The development of different stages was recorded during zygote-to-ookinete differentiation in three independent experiments. Most control parasites were transformed into ookinetes, but MLN4924-treated parasites failed to transition from stage I–II of zygote to ookinete differentiation. (**C**) Effect of MLN4924 on oocyst development in the mosquito midgut. Blood-stage parasites were treated with MLN4924 and used to feed mosquitoes. Six and 14 days later, the midguts were dissected, and the oocysts were counted. No oocysts were observed in mosquitoes fed MLN4924-treated parasites. Differential Interference Contrast (DIC) & Dimethyl sulfoxide (DMSO).

*Nedd8* KO parasites produced zygotes but not ookinetes (Fig. 2A), suggesting a defect in the process of zygote-to-okinete development. Therefore, we analyzed the different stages of zygote-to-ookinete differentiation using the P28 antibody (23). We found all the stages of parasites among the WT GFP strains during zygote-to-ookinete differentiation, whereas the *Nedd8* KO parasites exhibited small apical protrusions and were found to be developmentally arrested during the stage I–II transition (Fig. 2A; Fig. S3 at https://doi.org/10.6084/m9.figshare.25224743.v1). The NAE inhibitor MLN4924 has been shown to function as a potent and highly selective inhibitor of the NEDD8 system (24). We observed that the inhibition of neddylation with MLN4924 blocked zygote differentiation during the stage I–II transition (Fig. 2B). To understand malaria transmission dynamics, MLN4924-treated parasites were fed to mosquitoes using membrane glass feeders. We observed the development of oocysts in the mosquitoes fed control parasites but not in the MLN4924-treated parasite-fed mosquitoes (Fig. 2C). Like in the *Nedd8* KO population, MLN4924 treatment blocks parasite development during the stage I–II transition from zygote to ookinete differentiation, revealing the essential role of Nedd8 during this stage.

*Apicomplexan* parasites, including *Plasmodium* species, deviate significantly from the classical eukaryotic model and exhibit atypical cell division. The *Plasmodium* parasites undergo single meiotic and three prominent mitotic divisions throughout their life cycle. Neddylation activates Cullin-RING E3 ubiquitin ligases and regulates the SCF, which is critical for the cell cycle (2). Recently, it was demonstrated that the neddylation pathway is functional in malaria parasites and that cullins are Nedd8 substrates (20). However, to date, no experimental genetic approaches have been employed to validate the role of neddylation in the parasite life cycle. Here, for the first time, we functionally characterized the role of Nedd8 in the *P. berghei* life cycle using reverse genetics approaches. We deleted the *PbNedd8* gene and found that *Nedd8* KO parasites grow normally during erythrocytic stages and form gametocytes, which egress into gametes and fertilize to form zygotes while further failing to form ookinetes and transmit malaria.

In the malaria parasite, after fertilization, the zygote undergoes meiosis within 4 h (25). After 6–8 h of zygote development, an apical protrusion starts to form, followed by retorting, after which the resulting cells further transform into cigar-shaped ookinetes approximately 20 h postfertilization (26). Female-stored mRNAs facilitate this process, and long-term maintenance and stabilization of quiescent mRNAs depend on development of zygote inhibited (DOZI) (25). Parasites that lack DOZI fail to begin meiosis, and zygotes do not develop into ookinetes (25). Neddylation has been implicated in the assembly of stress granules. Blocking neddylation affects stress granule formation in a cullin-independent manner (27). However, whether DOZI or stress granule proteins in malaria parasites are neddylated needs further investigation. Another possible mechanism is that for the smooth translation of female-stored mRNAs, an optimal level of ribosomal proteins are needed, and a lack of neddylation affects this process. The ubiquitin-proteasome pathway maintains the optimal level of ribosomal proteins. CULs are core components of CRLs that regulate the degradation and subcellular trafficking of proteins. CULs are regulated through neddylation (2). In cultured silkworm cells, a lack of neddylation leads to cell cycle arrest at the G2/M phase and results in defects in chromosome congression and segregation (28). The authors found that the neddylation system can control multiple pathways in silkworms (28). We hypothesize that the recycling of ribosomal proteins via the neddylation process was possibly affected in Nedd8 KO parasites, which led to cell cycle arrest and apical protrusion formation; however, this hypothesis requires further investigation. Our findings are consistent with a recent report in *Trypanosoma brucei* showing that depletion of Nedd8 by RNAi abolishes global protein ubiquitination and causes defects in mitosis, spindle assembly, and chromosome segregation.

Taken together, the findings of the present study revealed the essential role of Nedd8 in *P. berghei*, and a lack of neddylation led to cell cycle arrest and the transformation of the zygote into an ookinete. This study could provide a basis for future investigations on cell cycle regulation in malaria parasites.
